# Competency and Role Development in Advanced Nursing Practice Following a Swiss Master’s Cohort Through Education and Early Clinical Practice: Protocol for a Longitudinal Convergent Mixed Methods Study

**DOI:** 10.2196/85773

**Published:** 2026-03-30

**Authors:** Astrid Braun, André Fringer, Heidrun Gattinger, Christian Eissler, Maya Zumstein-Shaha, Thomas Beer, Martina Roes

**Affiliations:** 1 School of Health Sciences, ZHAW Zurich University of Applied Sciences, Katharina-Sulzer-Platz 9, Winterthur, 8400, Switzerland, +41 58 934 43 41; 2 Department of Nursing Science, Witten/Herdecke University, Witten, Germany; 3 Institute of Health Sciences, Ostschweizer Fachhochschule OST, St. Gallen, Switzerland; 4 School of Health Professions, Bern University of Applied Sciences, Bern, Switzerland

**Keywords:** advanced practice nurse, advanced nursing practice, competence development, role development, mixed methods, longitudinal design, educational research

## Abstract

**Background:**

Master’s degree programs in advanced nursing practice have been established in Switzerland for approximately 2 decades, and the role of advanced practice nurses (APNs) is increasingly embedded within the Swiss health care system. Despite their growing presence, there remains a lack of clarity and consistency regarding this progress; persistent ambiguity in role definitions, expectations, and competencies continues to hinder consistent implementation and contributes to uncertainty for both APNs and employers. Existing research has not sufficiently examined how APN competencies and role perceptions develop during graduate education and the early transition to practice. This substudy of the *EDUCate* research initiative, *EDUCate*–Competence and Role Development (*EDUCate C-ARE*), focuses exclusively on a defined cohort of master’s students and follows their competence and role development longitudinally.

**Objective:**

The *EDUCate C-ARE* substudy aims to provide a comprehensive understanding of how APN competencies and role perceptions change during a master’s program and in the first year following graduation (research question [RQ] 1), identify factors that facilitate or hinder their development (RQ 2), and explore challenges that APNs report during this process (RQ 3).

**Methods:**

A longitudinal convergent mixed methods design will be applied. Quantitative data on APN competencies are collected using the Advanced Practice Nursing Competency Assessment Instrument at 4 time points (T0-T3), and qualitative data are generated through semistructured interviews at 3 time points (I1-I3). The quantitative and qualitative strands are analyzed independently and subsequently integrated using joint displays and narrative weaving to highlight convergence, complementarity, or divergence. Data collection spans 4 years, beginning in September 2023.

**Results:**

Baseline (T0) and midprogram (T1) quantitative data have been collected, with 141 students responding at T0 (full time: n=25, 17.7%; part time: n=56, 39.7%) and T1 (full time: n=17, 12.1%; part time: n=43, 30.5%). Data collection at T2 is ongoing. Initial analyses have been completed for T0 and T1. Qualitative interviews at I1 have been conducted and preliminarily analyzed (full time: n=6; part time: n=20). Data collection for full-time students is expected to conclude by May 2026, with preliminary findings anticipated by August 2026; for part-time students, data collection will continue until August 2027, with initial findings expected by October 2027. Integrated study results are anticipated by late 2026, with final analyses following completion of all data collection phases.

**Conclusions:**

This study will generate longitudinal evidence on APN competence and role development in a Swiss master’s cohort. By developing a conceptual, explanatory model of competence acquisition, role perception, and influencing factors, the study is expected to inform curriculum development, strengthen role clarity, and support the evidence-based implementation of APN roles in the Swiss health care system. Findings will contribute to national and international discussions on APN competency frameworks and professional role development.

## Introduction

### Background

Since the 1960s, advanced nursing practice (ANP) has been recognized as a key strategy for addressing global health care challenges and advancing health system development, with ANP programs implemented in more than 70 countries [1,2]. In particular, the integration of ANP in primary care has been promoted to improve access to care and respond to evolving population health needs [1,2]. The role of advanced practice nurses (APNs) was conceptualized to meet increasing demands for health care services while supporting professional development and career progression within nursing [2].

In Switzerland, universities of applied sciences and academic institutions have offered master’s degree programs in ANP for over 2 decades [3,4], awarding the Master of Science in Nursing degree [5]. A structured nursing development framework was established in collaboration with graduates of level I and II professional training programs [4,6,7]. In 2010, a total of 3 universities of applied sciences (Bern University of Applied Sciences [BFH], Eastern Switzerland University of Applied Sciences [OST], and Zurich University of Applied Sciences [ZHAW]) jointly developed a master’s program in nursing [8,9]. Since 2019, these institutions have independently offered the program with a variety of specializations.

### Conceptual Background and Role Expectations

The conceptual foundation of the APN role is rooted in the ANP framework. APNs are expected to apply the theoretical and evidence-based knowledge acquired during academic training; role development and implementation are shaped by national health care system structures and population needs [7,10]. Academic preparation generally involves a Master of Science in Nursing or an equivalent graduate qualification. The ANP framework includes several established advanced nursing roles, such as APNs, clinical nurse specialists, and nurse practitioners. These roles share a common foundation in advanced clinical expertise, evidence-based practice, and expanded professional responsibility while allowing for context-specific differentiation and adaptation [10]. They are characterized by competencies such as expert coaching and guidance, counseling and consultation, ethical decision-making, interdisciplinary collaboration, clinical and professional leadership, and evidence-based practice [10]. International efforts, including the 2020 International Council of Nurses APN guidelines, aim to strengthen coherence and clarity in the conceptualization and implementation of ANP and expand APN roles to meet evolving health care needs [11-17].

### Swiss Context: Opportunities and Ongoing Role Ambiguity

In German-speaking Switzerland, the APN role has become increasingly integrated into existing health care structures. APN competencies are recognized as a sustainable and essential contribution to current and future system challenges [18]. However, persistent role ambiguity, including variability in expectations within and across professional groups and context-dependent perceptions of the defined and self-defined role, has led to role conflicts, excessive demands, and insecurity, which have been identified as potential contributors to early attrition from the profession [1,8,13,14,19-26]. Studies demonstrate that insufficient role clarity and variability in role enactment contribute to role conflict, limited autonomy, and barriers to effective implementation [22,26].

Regulatory efforts by the Swiss association Advanced Practice Nursing Switzerland focus on minimum standards for certification and consistent training quality, including criteria governing access to the APN title to ensure that qualifications match the expanded scope of practice [27].

In parallel, the objectives defined by the Swiss Professional Association of Nurses provide a central reference point for educational institutions to align curricula with evolving system needs and anticipated employment opportunities, ensuring that graduates can be strategically integrated into the health care workforce [28,29]. Taken together, these developments underscore the importance of role clarity and structured competency development to support effective APN implementation in Switzerland. International research underscores that the transition from graduate education to early clinical practice is a critical phase for APNs, marked by shifting expectations, increasing responsibility, and role adaptation challenges [30,31]. Evidence indicates that targeted support during this transition is essential for role enactment, professional identity formation, and sustainable APN integration.

### Gap in the Literature

Despite progress in ANP education and role establishment, research on competence-oriented master’s programs in nursing remains limited, and systematic analyses that identify and evaluate the competencies acquired within such programs are lacking [32-37]. This gap constrains evidence-informed curriculum development and the strategic deployment of APNs within the Swiss health care system.

### Rationale and Scope of This Substudy

In 2021, BFH, OST, and ZHAW launched the overarching *EDUCate* initiative in German-speaking Switzerland.

This substudy of EDUCate–Competence and Role Development (*EDUCate C-ARE*) focuses exclusively on a defined cohort of master’s students to ensure feasibility within the 4-year study period.

It addresses the identified evidence gap by examining how higher education influences the development of competencies and professional roles among master’s students in ANP, with the aim of informing the strategic deployment of APNs within the Swiss health care system [8,38-41]

### Conceptual Definitions

In this study, 3 related but distinct concepts are used:

- “Role development” refers to the broader process through which APN roles evolve at the system and organizational level, such as through policy changes, workforce planning, and institutional role implementation. This concept is not the focus of this substudy.
- “Role acquisition” describes how individual nurses adopt and internalize the advanced practice role during and after their education. It reflects the process of gaining competencies and integrating the APN role into professional identity.
- “Role perception” refers to how nurses understand and interpret their own role and responsibilities within clinical practice at a given point in time. It captures subjective experiences and expectations related to the APN role.

These definitions guide the interpretation of findings and ensure conceptual consistency throughout the manuscript. They are grounded in established conceptual frameworks for APN roles [10,20].

### Study Aim and Objectives

The aim of the substudy is to longitudinally examine how APN competencies and role perceptions evolve among a defined cohort of master’s students during their education and into the first year of clinical practice in Switzerland.

To achieve this aim, the study pursues the following objectives:

1. To assess changes in APN competencies and role perceptions at 4 measurement points (T0-T3) during and after master’s education
2. To identify factors that facilitate or hinder competency acquisition and role development throughout the educational and early practice trajectory
3. To explore challenges experienced by APNs during this process as described by master’s students and alumni
4. To integrate quantitative and qualitative findings to provide a comprehensive understanding of APN development in the Swiss context

To address these objectives, the study is guided by the following research questions (RQs):

- RQ 1—in what ways do APN competencies and role perceptions change during the master’s program and in the first year following graduation?
- RQ 2—which factors facilitate or hinder the development of APN competencies and roles?
- RQ 3—what challenges do APNs report in relation to their professional development during this process?

## Methods

### Study Design

**Figure 1.**
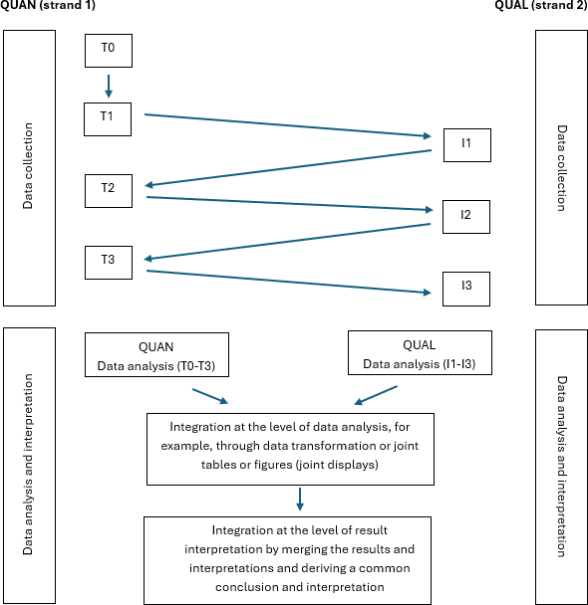
Longitudinal convergent mixed methods design with quantitative (QUAN) and qualitative (QUAL) measurement time points and subsequent data integration (own illustration). I1: midprogram; I2: program completion; I3: 1 year after graduation; T0: program start; T1: midprogram; T2: program completion; T3: after graduation.

This study uses a longitudinal convergent mixed methods approach in which the quantitative and qualitative components are conducted concurrently and independently [42,43]. Data from both strands are collected and analyzed separately to reduce the risk of mutual influence and preserve the distinct strength of each methodological approach. Figure 1 provides an overview of this parallel structure.

A defined cohort of master’s students (start year: 2023) will be followed across 4 quantitative measurement time points (T0: program start; T1: midprogram; T2: program completion; T3: after graduation) and 3 qualitative interview points (I1: midprogram; I2: program completion; I3: 1 year after graduation).

Quantitative data are collected using the Advanced Practice Nursing Competency Assessment Instrument (APNCAI) [44], whereas qualitative data are generated through semistructured interviews. This design allows for the examination of APN competency acquisition and role perception trajectories within the same cohort over time. After separate analyses, data from both strands are integrated to provide a comprehensive picture of APN development during education and into early clinical practice.

Data collection spans 4 years beginning in 2023, covering both the academic training phase and the transition to professional practice. This extended longitudinal period supports valid and meaningful analysis. Given the complexity of the research topic, the longitudinal convergent mixed methods design is particularly suited to capturing both broad patterns and in-depth experiences [42,45-48].

### Methodological Framework and Integration

The qualitative component follows the constructivist grounded theory approach by Charmaz [49], emphasizing iterative coding, constant comparison, and theory generation grounded in participants’ accounts. Quantitative and qualitative analyses are conducted independently. Integration occurs subsequently through joint displays and narrative weaving to identify convergence and complementarity.

The combination of methodological strands offers a complementary perspective: quantitative data provide measurable trends in competence development, whereas qualitative data offer detailed insights into individual experiences, contextual factors, and perceived challenges. The mixed methods design is particularly appropriate because the study involves diverse data types that capture both measurable trends (quantitative) and rich experiential accounts (qualitative). This combination allows for deeper explanatory insights, ensuring that quantitative patterns can be interpreted and contextualized through qualitative perspectives. Together, these approaches capture both measurable progression and the lived experience.

In line with Kuckartz [50] and international mixed methods scholarship [42,43,47], mixed methods are understood as the purposeful combination and integration of quantitative and qualitative approaches. Data from both strands are collected within the same overarching design, and integration typically occurs after completion of separate analyses.

### Methodological Rationale

To address the study’s complex RQs, a well-grounded methodological approach is essential [51]. The phenomenon under investigation is embedded in the understanding that education and learning processes occur continuously across the life span [52]. Individuals acquire knowledge and competencies at different stages, making a longitudinal mixed methods design appropriate.

The conceptualization by Gräsel [53] of empirical educational research provides a relevant framework for this study. Educational research examines learning processes, competence acquisition, influencing factors, and the effects of education on individuals and institutions. Its problem orientation, interdisciplinary character, and use of empirical methods align closely with the aims of investigating competency and role development in advanced nursing education.

### Setting and Participants

The study will be conducted at 3 universities of applied sciences in Switzerland: OST, BFH, and ZHAW. The target population comprises all students enrolled in the 2023 master’s cohort of APN programs at these sites. Cohort sizes vary by institution (approximately 12 in OST, 60 in BFH, and 60 in ZHAW). Students are enrolled in either full-time (FT) or part-time (PT) study modes. Measurement time points are mode adjusted: T1 takes place at the program midpoint (FT: 9 months; PT: 18 months), and T2 takes place at program completion (FT: 18 months; PT: 36 months). The inclusion criteria for participation in this study are membership in the 2023 cohort and provision of informed consent. Exclusion criteria include instances of nonconsent.

### Recruitment and Sampling

All eligible students in the 2023 master’s cohort are invited at program entry (T0) to participate in the quantitative surveys and to be recontacted at subsequent time points (T1-T3). For the qualitative component, interview participants are drawn from the same cohort at I1 to I3. Sampling for interviews aims to capture variation by site (OST, BFH, and ZHAW) and study mode (FT or PT). Participation is voluntary, with the option to withdraw at any time.

As of autumn of 2023, approximately 120 students had commenced their studies across the participating universities as part of the overarching *EDUCate* project. Recruitment of both master’s students and clinically active APNs will be coordinated through the heads of the respective degree programs and supported by a dedicated project website.

For the quantitative component, a target response of 15% to 20% is anticipated. The qualitative component follows the principles of grounded theory methodology, in which theoretical sampling, a core feature of this approach, guides the number and selection of interviews. Data collection continues until theoretical saturation is reached [54].

### Data Collection

#### Overview

**Figure 2.**
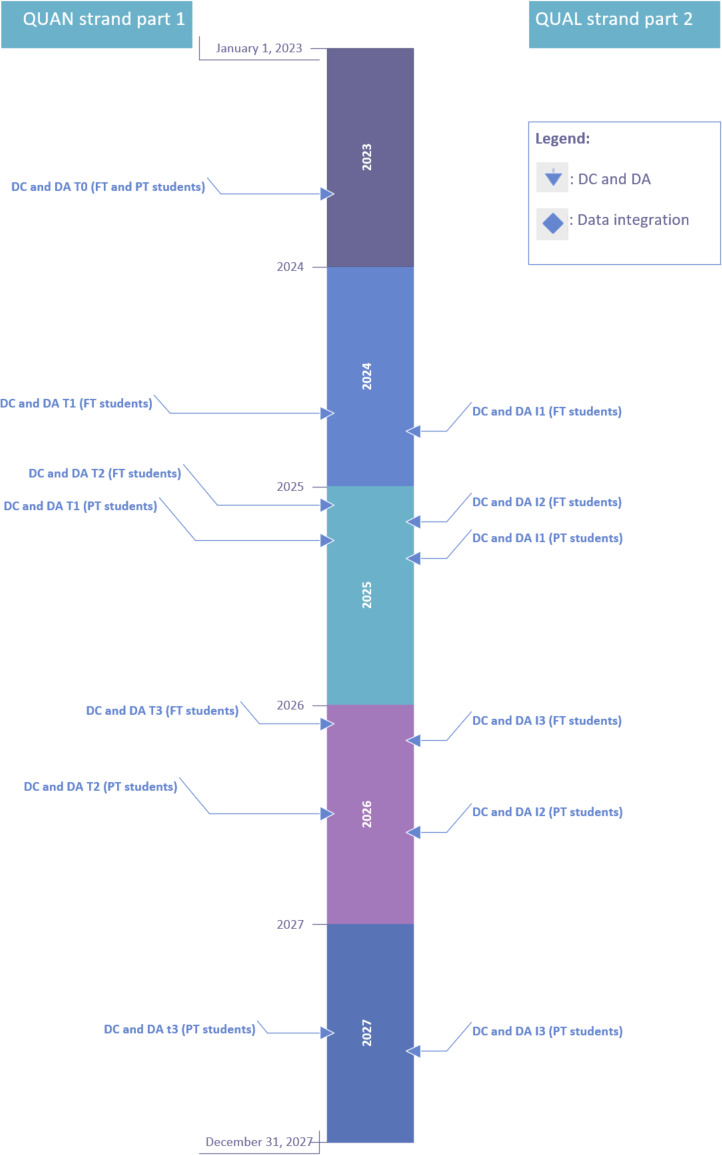
Overview of the timeline for data collection (DC), data analysis (DA), and integration. FT: full time; I1: midprogram; I2: program completion; I3: 1 year after graduation; PT: part time; QUAL: qualitative; QUAN: quantitative; T0: program start; T1: midprogram; T2: program completion; T3: after graduation.

In the *EDUCate C-ARE* study, data collection follows a longitudinal convergent mixed methods design in which quantitative and qualitative components are conducted sequentially across multiple time points (T0, T1, I1, T2, I2, T3, and I3), as illustrated in Figure 2. This design enables a comprehensive mapping of the development of APN competencies and role development over time.

Quantitative and qualitative data are collected and analyzed separately to ensure methodological rigor. The design provides both cross-sectional and longitudinal insights. When appropriate, comparisons with relevant subgroups (eg, PT students or other cohorts) may be conducted to identify patterns of convergence, divergence, or overlap.

#### Quantitative Data Collection

Quantitative data are collected using the APNCAI [44]. This 44-item instrument assesses core competencies of APNs across 8 dimensions: research and evidence-based practice, clinical and professional leadership, interprofessional relationships, mentoring, professional autonomy, quality and care management, professional education and teaching, and health promotion. Data are gathered from the selected student cohort at the 4 predefined time points: T0 (at the commencement of the master’s program), T1 (midway through the program; after 12 months for FT students or 16 months for PT students), T2 (at program completion), and T3 (12 months after graduation). Mode (FT or PT) and site (OST, BFH, or ZHAW) are recorded to enable subgroup summaries and comparisons.

The quantitative component (strand 1) of the study follows a longitudinal design with repeated measurement across 4 time points [42,55], enabling the assessment of changes in participants’ self-assessed competencies and role perceptions over time. This approach supports the identification of temporal developments and potential long-term effects of the educational program on participants’ professional growth and role understanding.

#### Qualitative Data Collection

Qualitative data are collected via semistructured interviews at 3 predefined phases: I1 (midprogram), I2 (program completion), and I3 (1 year after graduation). The interview guide is aligned with APN competency domains and focuses on role acquisition and role perception, as well as perceived facilitators, barriers, and challenges during education and transition to practice. Interviews are audio recorded and transcribed verbatim in accordance with data protection regulations and the transcription standards of Dresing and Pehl [56], including low-level transcription details [50]. Pretests of the interview guide were conducted with former student cohorts to ensure clarity and relevance. Transcription is performed by an internal transcription service of the university to ensure accuracy and data security. Interview guides are provided in Multimedia Appendix 1 [42,50,57-59].

The qualitative component (strand 2) is designed as a descriptive study using grounded theory methodology [54,60]. Semistructured interviews are conducted with a purposive sample of students and practicing APNs to explore their subjective experience of competence development and role formation. The interviews aim to elicit participants’ perceptions and expectations related to their academic and professional roles, explore how they experience their everyday work and study life, and identify factors that influence role performance and potential solutions to address challenges [57].

Participants are selected using a maximum variation sampling strategy (minimum and maximum contrast) to ensure heterogeneity within each cohort. Approximately 15% to 20% of students per cohort will be invited to participate [58]. To reach theoretical saturation, further recruitment may be necessary, with a target distribution of 50%, 30%, and 10% of interviews at I1, I2, and I3, respectively. Recruitment is coordinated by the respective program directors at the participating universities of applied sciences. All interviews are conducted online, recorded with participants’ consent, and stored securely on a protected network drive. Access to the data will be restricted to the first author and designated project staff.

### Outcomes

The primary quantitative outcome is the APNCAI total score over time (T0-T3). The secondary quantitative outcomes are APNCAI domain-level scores summarized overall and by study mode (FT or PT) and site (OST, BFH, or ZHAW). Primary qualitative outcomes are themes describing role acquisition and role perception across I1 to I3. Secondary qualitative outcomes are themes on facilitators, barriers, and reported challenges during the educational trajectory and early clinical practice.

### Quantitative Data Analysis

Quantitative data will be analyzed using R (version 4.5.0; R Foundation for Statistical Computing). Descriptive summaries (means, SDs, medians, and IQRs) are provided for total and domain scores at each time point. Visualizations such as box plots depict distributions and changes over time, including stratifications by study mode (FT or PT) and site (BFH, OST, or ZHAW). Where repeated measurements per individual are available, within-cohort change is examined using appropriate paired or repeated-measure approaches. Where only cross-sectional snapshots are available, between-group comparisons at each time point use appropriate parametric or nonparametric tests. Effect sizes and 95% CIs are reported where applicable.

Statistical procedures include descriptive statistics; ANOVA; Spearman rank correlation; and, where appropriate, linear mixed-effects models to account for repeated measures and individual-level variation over time. All statistical assumptions will be tested prior to analysis, including normality (eg, Shapiro-Wilks test), homogeneity of variances (Leven test), and sphericity (Mauchly test) for repeated-measure ANOVA. Residual diagnostics will be conducted for mixed models to ensure model validity.

Group comparisons explore differences between FT and PT students, as well as between cohorts from the 3 participating universities of applied sciences: BFH and ZHAW (each with approximately 55 students) and OST (with approximately 10 students). Due to unequal group sizes, appropriate statistical techniques are applied to ensure robustness. All participants hold a Bachelor of Science in Nursing, ensuring a consistent educational baseline across cohorts.

To uphold methodological rigor, the principles of objectivity, reliability, and validity are maintained throughout all stages of data collection and analysis. Objectivity is ensured through standardized procedures independent of the individual administering the survey. Reliability and validity are supported by implementing the instrument across multiple institutions. Pretests with earlier student cohorts were conducted to optimize the instrument and ensure its applicability. Data are securely stored and accessible only to authorized project staff.

### Qualitative Data Analysis

Qualitative data are analyzed using constructivist grounded theory (Charmaz) following iterative coding and categorization to develop a theoretical understanding of the studied phenomenon. The analysis includes initial coding, focused coding, constant comparison across cases and time points (I1-I3), and analytic memoing to trace category development. Classic coding steps (open coding, axial coding, and selective coding [61]) are applied. The MAXQDA 2025 software (VERBI GmbH) [50,62] is used to support data management, coding, and analysis.

The analysis seeks to build a conceptual understanding of role acquisition and role perception and explicate facilitators, barriers, and challenges across the educational and transition-to-practice trajectory. An audit trail and reflexive memos are maintained to enhance transparency and trustworthiness.

To ensure methodological rigor, the analysis will adhere to established qualitative quality criteria such as transparency, intersubjectivity, and comprehensiveness [63]. In addition, the study will follow the trustworthiness criteria proposed by Lincoln und Guba [64], including credibility, transferability, dependability, and confirmability, to further strengthen the robustness and transparency of the qualitative component. Transparency is ensured through detailed documentation of all analytical steps and the use of software-supported coding. Intersubjective comprehensibility is promoted through regular team discussions and reflective engagement with the data. The aim is to develop a theoretically grounded and context-sensitive understanding of APN role development from the participants’ perspectives.

### Integration of Findings and Data Integration

Integration of findings is conducted after the completion of both quantitative and qualitative analyses. A sequential approach is applied in which results from each strand are first analyzed independently and then brought together during the interpretation phase. Joint displays and narrative weaving are used to relate quantitative patterns in APNCAI scores (T0-T3) to qualitative themes (I1-I3) on role acquisition, role perception, and contextual factors. Integration is guided by the study objectives and RQs, highlighting convergence, complementarity, or divergence between strands.

Data integration represents a central component of the mixed methods approach. In accordance with best practices, quantitative and qualitative data will be analyzed separately using methodologies appropriate to each paradigm. Following the completion of data collection and initial analyses, the findings will be brought together through a process of integration by triangulation. This approach involves systematic comparison and synthesis of results from both strands to enhance the validity, complementarity, and depth of interpretation.

Visual representations such as joint displays and matrices are used to facilitate the integration process and support interpretation and reporting. The integration strategy is grounded in the principle of methodological triangulation, which leverages the strengths of both quantitative and qualitative approaches to provide a more comprehensive understanding of the research problem [65]. This process also serves to mitigate the limitations inherent to each individual method by enabling cross-validation and complementary insights [66].

Integration focuses on identifying areas of convergence, divergence, and expansion between the 2 data types. Quantitative results contribute to generalizability, whereas qualitative findings will offer a contextualized, in-depth understanding.

Following the framework proposed by Creswell and Plano Clark [42], this integrative process culminates in the development of meta-inferences, which are overarching conclusions that emerge from the synthesis of both data strands. These meta-inferences aim to generate insights that are more robust, nuanced, and meaningful than those derived from a single method, thereby strengthening the overall explanatory power of the study.

### Reporting Standards and Methodological Quality

The reporting of this protocol and the subsequent mixed methods study is guided by internationally recognized standards to ensure methodological rigor, transparency, and clarity. Reporting follows the GRAMMS (Good Reporting of a Mixed Methods Study) [47,52] framework for mixed methods research. For the qualitative components, the SRQR (Standards for Reporting Qualitative Research) guidelines will be applied [67]. For the observational quantitative elements, the STROBE (Strengthening the Reporting of Observational Studies in Epidemiology) guidelines will be used [68].

To support methodological coherence and quality across both strands, the Mixed Methods Appraisal Tool [69] and the GRAMMS guidelines [47,52] are used throughout the design, analysis, and integration phases. These tools were selected because they are widely accepted in health research and are recommended by the EQUATOR (Enhancing the Quality and Transparency of Health Research) Network [70], which provides central guidance for best practices in research reporting.

Methodological quality is further ensured through strategies such as maintaining an audit trail, reflexive memoing, and peer debriefing during qualitative analysis. Data collection and analysis procedures are standardized across participating sites to enhance consistency and reliability.

### Ethical Considerations

The overarching *EDUCate* study has been reviewed by the Ethics Committee of the Canton of Zurich, Switzerland, and was deemed exempt from formal authorization in accordance with the Swiss Human Research Act (Business Administration System for Ethics Committees Req-2019-00875). This substudy is conducted within the approved *EDUCate* framework.

Participation in both the survey and interview components is voluntary, and individuals who choose not to participate will not experience any disadvantages or negative consequences. Written informed consent is obtained from all participants prior to data collection.

The study is being conducted in full compliance with the Swiss Human Research Act [71], applicable Swiss legislation on data protection and research involving human participants [72], the Declaration of Helsinki [73], and the principles of good clinical practice [74]. All procedures related to confidentiality, data protection, and participant rights are strictly adhered to throughout the study. No financial or material compensation is provided to participants for their involvement in this study.

## Results

### Overview

The *EDUCate C-ARE* study is internally funded by ZHAW in June 2023. Data collection began in September 2023 with the cohort starting the master’s program in ANP in the same year.

Quantitative data collection at baseline (T0) and midprogram (T1) has been conducted for the 2023 cohort. In total, 81 students completed T0 (FT: n=25, 30.9%; PT: n=56, 69.1%), and 60 students completed T1 (FT: n=17, 28.3%; PT: n=43, 71.7%).

Data collection at T2 (program completion) is ongoing, with 15 responses received to date (FT: n=15, 100%); PT and additional FT responses are pending. Final numbers will be reported once data collection is complete.

### Quantitative Data Collection Timeline

For FT students, T0 is September 2023, T1 is September 2024, T2 is January 2025, and T3 is January 2026. For PT students, T1 is April 2025, T2 is May 2026, and T3 is May 2027.

### Qualitative Interview Timeline

For FT students, I1 is October 2024, I2 is February 2025, and I3 is February 2026. For PT students, I1 is May 2025, I2 is June 2026, and I3 is June 2027.

Preliminary qualitative analyses have been completed for the I1 interviews.

### Overall Study Progress and Preliminary Findings

Initial quantitative analyses have been completed for T0 and T1. Data collection for FT students is expected to be completed by May 2026, with preliminary results anticipated by August 2026. For PT students, data collection is projected to conclude by August 2027, with initial findings expected by October 2027. Final analysis and integrated results will follow the completion of all data collection phases. The first overarching study results are planned to be available by the end of 2026.

## Discussion

### Principal Findings

The primary objective of the *EDUCate C-ARE* substudy is to document and analyze the development and application of competencies, as well as the evolving roles of master’s students and clinically active APNs. Guided by RQs 1 to 3, the study is expected to yield novel insights into the representation of professional competencies, role expectations and enactment, and the perceived benefits and challenges associated with APN roles over the program and the first year in practice. Particular attention will be paid to the clarity or ambiguity of role definitions and the implications for professional identity and practice. The study further aims to contribute to the international comparability of APN competencies and role profiles [75].

A central anticipated outcome is the formulation of a conceptual, explanatory model that outlines barriers to competency and role development and proposes feasible strategies to address them. In doing so, the study seeks to deepen understanding of APN competencies and role trajectories and underscore the importance of role clarity and professional development for the advancement of nursing practice and the effectiveness of educational programs. These insights are expected to inform academic curricula and clinical practice and support the strategic implementation of APN roles in health care systems.

### Comparison With Prior Work

The study’s objectives and anticipated contributions are situated within established frameworks and evidence. Alignment with the model of ANP by Hamric [76] provides a conceptual basis for examining advanced competencies, role expectations, and professional identity. The use of the APNCAI enables a connection with existing validation studies and supports international comparability of competence profiles [44]. In the Swiss context, where empirical evidence on APN role development remains limited, this study addresses a documented gap by offering longitudinal, mixed methods evidence on competency acquisition, role perception, and influencing factors. In combination, these anchors situate the expected findings within the broader international APN discourse while responding to national needs for role clarification and evidence-informed implementation.

### Strengths and Limitations

The study’s convergent mixed methods design, applied longitudinally across 4 quantitative time points and 3 qualitative interview phases, allows for the examination of change over time and the integration of breadth and depth of evidence. The combination of a standardized competence assessment (APNCAI) with constructivist grounded theory supports cross-method triangulation and enhances credibility, validity, and depth of interpretation. Data collection across multiple universities of applied sciences increases heterogeneity and supports external validity. The explicit integration strategy (eg, joint displays and narrative weaving) strengthens meta-inference and theory development and constitutes a methodological contribution for the study of APN role and competence development.

Several limitations must be acknowledged. The mixed methods design is resource intensive and constrained by time and participant availability. The sample is limited to German-speaking regions of Switzerland, which may affect the generalizability to other linguistic or cultural contexts. Additional constraints include potential language and professional barriers, limited financial and temporal resources, and the risk of selection bias as individuals with a preexisting interest in APN role development may have been more likely to participate.

### Conclusions

The *EDUCate C-ARE* study is expected to provide relevant insights into the development of competencies and roles among master’s students and practicing APNs in Switzerland. Through methodological triangulation and the development of a conceptual explanatory model, the study aims to contribute to both theoretical advancement and practical innovation in the ANP field. By identifying key barriers and proposing actionable solutions, it seeks to support targeted improvements in education, clinical practice, and professional development and reinforce the critical contribution of APNs to the evolving health care landscape.

### Dissemination

The findings of the *EDUCate C-ARE* study will be disseminated through publications in national and international peer-reviewed journals. In addition, the validated APNCAI assessment instrument will be translated following established, evidence-based guidelines for the translation of research and clinical instruments. A dedicated project website has been developed to provide background information and will be regularly updated with new research findings and outputs [77].

## Supplementary material

10.2196/85773 Multimedia Appendix 1 Interview guide.

## Abbreviations

ANP: advanced nursing practice
APN: advanced practice nurse
APNCAI: Advanced Practice Nursing Competency Assessment Instrument
BFH: Bern University of Applied Sciences
EDUCate C-ARE: EDUCate–Competence and Role Development
EQUATOR: Enhancing the Quality and Transparency of Health Research
FT: full time
GRAMMS: Good Reporting of a Mixed Methods Study
OST: Eastern Switzerland University of Applied Sciences
PT: part time
RQ: research question
SRQR: Standards for Reporting Qualitative Research
STROBE: Strengthening the Reporting of Observational Studies in Epidemiology
ZHAW: Zurich University of Applied Sciences
