# Supplementary material for: Competency and Role Development in Advanced Nursing Practice Following a Swiss Master’s Cohort Through Education and Early Clinical Practice: Protocol for a Longitudinal Convergent Mixed Methods Study
Source: JMIR Res Protoc. 2026 Mar 30;15:e85773. doi: 10.2196/85773 (PMC13035200; doi:10.2196/85773)
Supplement: Multimedia Appendix 1 [file resprot-v15-e85773-s001.pdf]

Interviewleitfaden

Für halbstrukturierte Interviews i1-i3 im Rahmen des Projekts EDUCate

Fachhochschulübergreifende Edukations- und Begleitforschung

Arbeitsdokument, intern

Erstellt von Astrid Braun

Inhaltsverzeichnis

Terminologie und Theorieansatz 3
1.1 Fragestellung Proposal (Stiftung Pflegewissenschaft / SNF) 3
1.2 Qualitative Datenerhebung und -analyse 3

Interviewvorbereitung und Feldzugang 4

Durchführung Interviews i1-i3: Intro, Themenblöcke und Fragestellungen 5
3.1 Begrüssung, Kontext, Ziel und Aufbau des Interviews 5
3.1.1 Demografics 6
3.1.2 Einstieg: Über die Person / (Icebreaker) 8
3.2 Themenblock i1 (nach t1, Studienmitte) 8
Dimension 8: Gesundheitsförderung 9
Dimension 7: Ausbildung und Berufsbildung 9
Dimension 2: Klinische und fachliche Führung 10
Dimension 1: Evidenzbasierte Forschung und Praxis (EBFP) 10
3.3 Themenblock i2 (nach t3, 12 Monate nach Studienabschluss) 10
Dimension 3: Berufliche Selbständigkeit (professional autonomy) 11
Dimension 4: Interprofessionelle Beziehungen und Mentoring 11
Dimension 5: Qualitätsmanagement 11
Dimension 6: Pflegemanagement (Leadership) 11
3.4 Themenblock i3 (vor t4, mehr als 12 Monate und bis zu 3 Jahre nach Studienabschluss) 12
Themenblock A – Ausübung der APN-Rolle (und Rollenidentifikation – was und wie, Praktiken, Routinen, Zusammenarbeit mit anderen Professionen, Kommunikation) 12
Themenblock B – Entwicklung der Kompetenzen/Fähigkeiten (Skills) 12
Themenblock C – Vorbereitung auf APN-Rolle durch Studium (Kompetenzentwicklung / Ziel Studium evaluieren / weiterentwickeln) 12
Abschliessende Fragen (Selbsteinschätzung / Bryant-Lukosius et. al.; 2016) 13
3.5 Abschluss: Offene Fragen oder Anmerkungen, Dank und Verabschiedung 13

Interviewnachbereitung 14

Literaturverzeichnis 15

Terminologie und Theorieansatz

Für eine umfassende Beantwortung der Fragestellung, wird der Studie ein Convergent Parallel Mixed Methods Longitudinal Design zugrunde gelegt (Creswell & Plano Clark, 2017). Über einen Zeitraum von **vier Jahren** soll die Rollenerwartung sowie -entwicklung (qualitativ) und die Kompetenzentwicklung (quantitativ) beschrieben, analysiert und miteinander synthetisiert werden. Die geplante Zeitdauer wird für diesen Prozess als sinnvoll und notwendig erachtet, um den zeitlichen Entwicklungshorizont erfassen zu können.

Für ein angemessenes **Sample** wird diese Studie mit unterschiedlichen MSc-Studierendenkohorten durchgeführt. Die Rekrutierung erfolgt über die Studiengangsleitenden. Es wird von einer erwarteten Kohortengrösse von N= 120 ausgegangen (BFH n= 60, OST n=10 und ZHAW n=50).

Zur **Datensammlung** werden für den jeweiligen Untersuchungsstrang eigene spezifische Methoden eingesetzt.

1.1 Fragestellung Proposal (Stiftung Pflegewissenschaft / SNF)

Wie stellt sich die APN-Kompetenzentwicklung, die APN-Rollenentwicklung und -umsetzung von Studierenden und Alumni der MSc-Studiengänge in sowie bis zu drei Jahren nach deren Abschluss an den Hochschulen (BFH, OST und ZHAW) dar und welche *Erfahrungen* werden dabei gemacht?

- *Wie* entwickeln sich die APN-Kompetenzen und *wie* die APN-Rolle im Laufe der Ausbildung und den ersten drei Jahren nach MSc-Abschluss?
- *Welche* Herausforderungen lassen sich identifizieren und welche förderlichen und hinderlichen Faktoren zur Kompetenz- und Rollenentwicklung zeigen sich?
- *Welche* gesundheitspolitischen, ausbildungsbezogenen und institutionellen Massnahmen für die zukünftige Etablierung der APN-Rolle wären hilfreich?
- *Welche* förderlichen und hindernden Rahmenbedingungen lassen sich bei der Kompetenz- und Rollenentwicklung identifizieren?

1.2 Qualitative Datenerhebung und -analyse

Um die subjektiven Sichtweisen sowie das explizierbare Hintergrundwissen der Studienteilnehmenden (TN) zu erfassen, werden studien- sowie berufsbegleitende **Leitfadeninterviews** (Helfferich, 2014) durchgeführt. Dabei soll erschlossen werden, wie die TN ihren Studien- respektive Arbeitsalltag erleben, *wie* sie ihre Rolle ausüben, *welche* Erwartungen von *wem* an sie herangetragen werden und *wie* sie mit diesen Erwartungen umgehen. Ebenfalls von Interesse ist, *welche* Studieninhalte und *welche* organisatorischen/institutionellen Rahmenbedingungen förderlich oder hinderlich bei ihrer Rollenausübung wirken, *worin* sie günstige Bedingungen bzw. Probleme sehen und *was* sie als wünschenswerte Lösungen erachten. Das gezielte TN-Sampling erfolgt nach dem Prinzip der maximalen und minimalen Kontrastierung (Kelle & Kluge, 2010). Die theoretische Sättigung und der geplante Studienumfang erfordert bis zu 30 TN. Dazu werden die TN zur Bildung des heterogenen Samples entsprechend den Zeitpunkten (i1-i3) akquiriert. Angestrebt werden ca. 10 Interviews/Kohorte über alle drei Hochschulen. Die Interviewdurchführung findet online statt, um einerseits mit den Studierenden die Phasen ihres Wissenserwerbes, die Studienbedingungen und -strukturen zu besprechen und andererseits sollen hierbei studentische Ideen und Vorstellungen, die sie an die curriculare APN-Weiterentwicklung richten, identifiziert werden. Die TN-Interviews werden transkribiert und das Datenmaterial gemäss Grounded Theory-Methodologie analysiert (Strauss & Corbin, 1996). Das Management, die Datenanalyse sowie die Nachvollziehbarkeit des Analyseprozesses werden mit der Software MAXQDA 20.3 unterstützt und gesichert (Kuckartz, 2014).

Anschliessend werden die Ergebnisse der qualitativen und quantitativen Analyse integrativ zusammengeführt. In standortübergreifenden Sitzungen werden die Resultate daraufhin befragt, *ob* und *wie* sie sich entsprechen, ergänzen oder widersprechen. Dies erfolgt mit der Zielsetzung, einer

APN-Kompetenz- und APN-Rollen-Typologisierung, zu kontextualisieren, zu plausibilisieren und gegebenenfalls zu generalisieren, um die künftige schweizerische APN-Ausbildung zu planen sowie deren Rollenprofile zu entwerfen.

Die Studienteilnahme ist freiwillig. Die Studiendurchführung orientiert sich an den gesetzlichen schweizerischen Bestimmungen, der Helsinki-Deklaration sowie den Good Clinical Practice-Prinzipien.

Interviewvorbereitung und Feldzugang

- Rahmenbedingungen: Onlineinterviews (Zoom/teams)
  Mit Teams wurden in anderen Projekten bereits gute Erfahrungen gemacht. Die Terminfindung wurde durch die Onlinedurchführung leichter und die Interviews konnten gleich aufgezeichnet werden. Die Rückmeldungen waren positiv.
- Bei Terminvereinbarung hinweisen auf:
  - Einwilligungserklärung: keine erforderlich, die Bereitschaft am Interview teilzunehmen wird als Zusage (Informed Consent) angesehen.
  - Anonymisierung / Pseudonymisierung und ggf. Autorisierung der Aussagen von Personen mit bestimmten Funktionen bzw. Arbeitsorten (bspw. Leitung/MediX Gemeinschaftspraxis), die Rückschlüsse auf deren Identität zulassen
    Bei Experteninterviews, aber auch bspw. Zeitzeugen-Interviews ist die Anonymisierung/Pseudonymisierung ebenfalls nicht immer möglich oder sinnvoll. Hier empfiehlt es sich, die explizite Zustimmung zur Verarbeitung der personenbezogenen Daten einzuholen (Autorisierung der Aussagen).
  - Recording bei Präsenzinterviews: Smartphone, Aufnahmegeräte (Ersatzakkus, Powerbank u/o Batterien, Ladekabel, etc. – persönliche Vorbereitung)
  - Geplante Interviewdauer: 45-60 Minuten
- Interviewfragen werden *nicht* vorgängig an Gesprächspartner:in zur Vorbereitung abgeben
- Feldzugang: Erfolgt über Mailanfragen und Aufrufe an Studiengangskonferenzen
- Sprache des Interviews: CH- und/oder Schriftdeutsch
- Fragebogen in Print / Notizpapier bzw. elektronisch

Durchführung Interviews i1-i3: Intro, Themenblöcke und Fragestellungen

Zielgruppe:
- MSc-Studierende TZ und VZ-Modus (BFH, OST, ZHAW)
- Alumni (Post-MSc)

Setting / Kontext / Ziele:
Erleben des Studien- respektive Arbeitsalltag
- *wie* sie ihre Rolle ausüben,
- *welche* Erwartungen von *wem* an sie herangetragen werden und *wie* sie mit diesen Erwartungen umgehen.

Zielgruppe Studierende (i1-i2):
- *welche* Studieninhalte und *welche* organisatorischen/institutionellen Rahmenbedingungen förderlich oder hinderlich bei ihrer Rollenausübung wirken, *worin* sie günstige Bedingungen bzw. Probleme sehen und
- *was* sie als wünschenswerte Lösungen erachten.

Zielgruppe Post-MSc (Alumni) (i3):
- APN Rollen- und Kompetenzentwicklung

Formales
- Einwilligungserklärung (Pseudonymisierung/Autorisierung der Aussagen von Personen mit bestimmten Funktionen, die Rückschlüsse auf deren Identität zulassen)

3.1 Begrüssung, Kontext, Ziel und Aufbau des Interviews

Ich freue mich sehr, dass du/Sie sich heute für das Interview Zeit genommen haben und uns Einblicke in Ihre Erfahrungen als MSc-Studierende / APN zu ermöglichen. Mein Name ist xxx, ich bin (externe) wissenschaftliche/r Mitarbeiter:in (bzw. eigene Funktion/Rolle) einfügen an der Hochschule xxx und arbeite im Projekt EDUCate mit. Vielen Dank für deine/Ihre Bereitschaft, das Interview mit mir zu führen.

Im Forschungsprojekt interessieren uns:
1. Deine/Ihre Rollenerwartung die an dich/Sie herangetragen werden und die Rollenausübung
2. Organisatorische/strukturelle Rahmenbedingungen, die förderlich / hinderlich wirken
3. Mögliche Lösungsansätze

Dabei orientieren wir uns an den von Hamric et al. (2013) im APN-Modell beschriebenen Kernkompetenzen und fokussieren uns in diesem Interview auf vier der acht Kernkompetenzen.

Im heutigen Interview möchte ich mit dir/Ihnen über folgende Themenbereiche sprechen:
(1) Rollenerwartungen und Rollenausübung
(2) Deine/ Ihre Erlebnisse und Erfahrungen mit der Advanced Practice Nurse-Rollen

Wir sind im Projekt und auch bei Publikationen dazu verpflichtet, alle Daten vertraulich zu behandeln. Aus diesem Grund werden wir eine Anonymisierung / Pseudonymisierung durchführen bzw. Aussagen von Ihnen/Dir autorisieren zu lassen. Die Daten werden sicher und nur Projektmitarbeitende zugänglich aufbewahrt.
Zeitlicher Rahmen für das Interview nochmals abklären (ggf. Verlängerung möglich?).

Ich starte nun die Aufnahme.

3.1.1 Demografics

Vorname / Name:	
Datum Interview:	
Ort des Interviews:	☐ online (Teams/Zoom)<br>☐ physisch (Ort ergänzen):
Zeit (Beginn / Ende) des Interviews:	
Dauer des Interviews (h:min)	
Interviewer_in (Name ergänzen)	
Alter in Jahren	
Geschlecht / Gender:	☐ weiblich<br>☐ männlich<br>☐ Anderes, und zwar:_______________
Hochschule, an der das Studium absolviert wurde:	☐ Berner Fachhochschule (BFH)<br>☐ Ostschweizer Fachhochschule (OST)<br>☐ Zürcher Hochschule für Angewandte Wissenschaften (ZHAW)<br>☐ Anderes, und zwar: _______________
Vertiefung im Studium:	☐ Clinical Nurse Specialist (CNS)<br>☐ Nurse Practitioner (NP)<br>☐ Psychiatric Mental Health Nurse Practitioner (PMHNP)<br>☐ Forschung<br>☐ Kooperationsstudiengang (BFH/OST/ZHAW)<br>☐ Anderes, und zwar:_______________
Abschlussjahr:	
Studienmodus:	☐ Vollzeit<br>☐ Teilzeit<br>☐ Wechsel während dem Studium (Vollzeit auf Teilzeit)
Abschlussnote:	Note:<br>☐ keine Angabe
Berufserfahrung als APN in der klinischen Praxis (Anzahl Jahre ergänzen):	______________Jahre
Umfang absolvierter Weiterbildungen (begonnen u/o abgeschlossen innerhalb der letzten 5 Jahre, neben dem MSc-Studium Pflege)	☐ keine<br>☐ bis 450 Lernstunden, bspw. Certificate of Advanced Studies (CAS)<br>☐ 900 bis 1800 Lernstunden, bspw. Diploma of Advanced Studies (DAS), Master of Advanced Studies (MAS)<br>☐ mehr als 1800 Lernstunden (bspw. Masterstudium)<br>☐ Anderes, und zwar:_______________
Themenbereiche der begonnen / absolvierten Weiterbildungen innerhalb der letzten fünf Jahre:	☐ Klinische Pflegepraxis<br>☐ Leadership / Management<br>☐ Forschung / Wissenschaft

	☐ Anderes, und zwar:_______________
Geplante Weiterbildungen in den nächsten zwei Jahren	☐ keine<br>☐ bis 450 Lernstunden, bspw. (CAS)<br>☐ 900 bis 1800 Lernstunden, bspw. (DAS), (MAS)<br>☐ mehr als 1800 Lernstunden (bspw. Masterstudium)<br>☐ PhD (Promotion)<br>☐ Anderes, und zwar:_______________
Wenn Weiterbildung in den nächsten zwei Jahren geplant, welche Themenbereiche interessieren:	☐ Klinische Pflegepraxis<br>☐ Leadership / Management<br>☐ Forschung / Wissenschaft<br>☐ Anderes, und zwar:_______________<br>☐ Weiss nicht
Aktuelle Tätigkeit als APN (evtl. definieren)	☐ Ja<br>☐ Nein
Wenn ja: Stellenprozente in aktueller Anstellung:	______________%
Wenn ja: Setting:	☐ Akutspital<br>☐ Ambulanter Bereich<br>☐ Spitex<br>☐ Arztpraxis / Gemeinschafts-/Gruppenpraxis<br>☐ Langzeitpflege<br>☐ Psychiatrie<br>☐ Anderes, und zwar: _______________
Arbeitgeber und Kanton:	
Wenn ja: APN-Rollenausrichtung, die am besten zur aktuellen Anstellung passt:	☐ CNS<br>☐ NP<br>☐ Forschung<br>☐ Anderes, und zwar: _______________<br>☐ Weiss nicht
Hat die neue Rolle/Funktion eine Auswirkung auf deinen Lohn resp. eine bessere Vergütung zur Folge?	☐ ja<br>☐ nein<br>☐ unbekannt<br>☐ keine Angabe
Wenn ja: aktueller Verdienst:	☐ ≤ CHF 3000<br>☐ CHF 3001-4500<br>☐ 4501-6000<br>☐ CHF 6001-7500<br>☐ CHF 7501-9000<br>☐ ≥ CHF 9001<br>☐ keine Angabe

Selbsteinschätzung (CNS – NP-Kontinuum) (s. Dokument EDUCATE-Selbsteinschätzung CNS-NP-Kontiuum.pptx)

1. Wo sehen Sie sich/siehst du dich heute in Bezug auf die Ausübung Ihrer/deiner APN-Rolle? (blau)
2. Wohin möchten Sie sich/möchtest du dich selbst hin entwickeln? (CNS – NP-Kontiuum) / Zukunftspläne? (grün)
3. Wo haben Sie/hast du in Bezug auf die Ausübung deiner APN-Rolle gestartet? (rot)

Figure 1. Continuum of advanced practice nursing roles

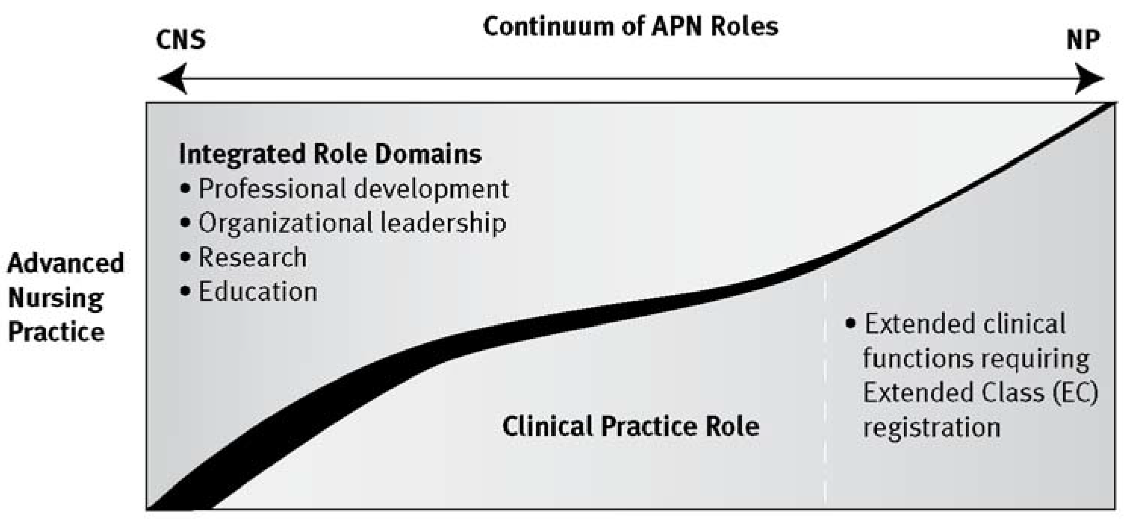

CNS= Clinical nurse specialist
Bryant-Lukosius, D. 2004 and 2008. *The Continuum of Advanced Practice Nursing Roles.* Unpublished document.

Vor Interview-Ende:<br>Am Ende des Interviews Interesse an einem Folgeinterview erfragen<br>(Messzeitpunkt i2, i3, ggf. i4).	☐ Ja, Mailadresse (privat) erfragen: _______________<br>☐ Nein

3.1.2 Einstieg: Über die Person / (Icebreaker)

Bitte schildern Sie mir kurz Ihre aktuelle Tätigkeit und Ihren Ausbildungsabschluss. (Hintergrundinformationen zur Person: Stand Studium, Ausbildungsgrad, Berufssituation/jetzige Tätigkeit)? → Unterscheidung zwischen Studierenden, APNs (oder nur via *Eckdaten?)*

3.2 Themenblock i1 (nach t1, Studienmitte)

Einstiegsfrage: Erzählen Sie bitte, wie Ihre aktuelle berufliche Tätigkeit aussieht.
APN-Rolle - neu übernommen? ggf. berufliche Veränderungspläne umgesetzt?

1. Welche Kompetenzen können Sie in Ihrer derzeitigen Tätigkeit ausüben?
2. Welche Hauptaufgaben haben Sie?

- In welchem Fachgebiet und Setting? (Fragen werden teilweise bereits bei den «Demografics» erhoben)

Infos für Interviewführende Person: Der APNCAI beinhaltet 8 Dimensionen davon werden während dem Interview i1 und Interview i2 jeweils 4 Dimensionen erhoben (gelb Interview i1 / türkis Interview i2).

1. Evidenzbasierte Forschung und Praxis
2. Klinische und fachliche Führung
3. Berufliche Selbständigkeit
4. Interprofessionelle Beziehungen und Mentorin
5. Qualitätsmanagement
6. Pflegemanagement
7. Ausbildung und Berufsbildung
8. Gesundheitsförderung

Dimension 8: Gesundheitsförderung

Einleitung: Bspw. Wir beginnen mit dem Thema Gesundheitsförderung.

1. Welche Prioritäten / Fokus haben Sie / hast du als APN, im Rahmen Ihrer/deiner APN-Tätigkeit? (primäre pflegerische Zielsetzung)
    a. Was ist Ihre/deine primäre pflegerische Zielsetzung als APN?
    b. Wie fördern Sie/du die Fähigkeiten im Selbstmanagement von Patient:innen / Klient:innen? (bspw. Strategien, die angewendet werden)
2. Was machen oder tun Sie/du um Ihre/deine eigene Gesundheit zu fördern (Eigenwahrnehmung)?
    a. Welche Massnahmen / Aktivitäten wenden Sie selbst an, um Ihre/deine Gesundheit zu fördern?
    b. Welche Massnahmen u/o Tätigkeiten verbinden Sie mit Gesundheitsförderung?
3. Erzähle(n) Sie mir, wie Sie/du in Ihrer/deiner beruflichen Setting mit Patient:innen, Klient:innen Gesundheitsförderung umsetzt?
    a. Welche Massnahmen, Aufgaben setzen Sie bei Patient:innen / Klient:innen etc. um/ein?
4. Welchen Anteil nimmt die Beratungspraxis in Ihrer/deiner klinischen Tätigkeit ein (aus Dimension 2/Beratung)

Dimension 7: Ausbildung und Berufsbildung

Überleitung: Sie studieren bereits ein Jahr Pflege im MSc-Programm.

1. Erzähle mir wie du lernst / deine Lernstrategien
   Vertiefungsfragen erst stellen, wenn bisher noch keine Antworten zu den Unterfragen gekommen sind
    a. in Bezug auf das Studium und Ihre Praxis
    b. Wie fördern Sie/du effektives Lernen?
    c. Wie sieht für Sie/dich eine optimale / förderliche Lernumgebung aus, in der Sie/du die AP-Kompetenzen ausbilden können?
    d. Was waren/sind Ihre Erfahrungen (Learning Lessons) im vergangenen Jahr? (bspw. anhand von Beispielen/Situationen die passiert sind)
2. Kannst du mir anhand einer Praxissituation schildern, wie du dein im Studium erworbenes Wissen angewendet hast.
    a. Welches erworbene Wissen wurde auf die Praxissituation übertragen?
    b. Hat sich dadurch etwas verändert und falls ja, was?
3. Interdisziplinäre Module (Studiumsinhalte): Welche Erfahrungen haben Sie zu / mit diesem Angebot gemacht! (nicht an OST, geplant ab HS24)
    a. Schildern Sie wie Sie/du im klinischen Alltag die klinische interprofessionelle Zusammenarbeit leben/erleben (Bezug zur Dimension 1)

Dimension 2: Klinische und fachliche Führung

Überleitung: Wie wechseln zur Thematik Leadership.

1. Das Hamric Modell sieht eine Leadership-Funktion vor, können Sie mir erzählen, wie sie diese Funktion ausüben.
    a. Welche Erfahrungen haben Sie zu/mit Leadership gemacht?
    b. Beschreiben Sie uns, wie Sie Leadership in ihrem Berufsalltag erleben und praktizieren/umsetzen!

Dimension 1: Evidenzbasierte Forschung und Praxis (EBFP)

Zum Abschluss interessiert mich noch:

1. Das Hamric Modell sieht vor, dass APN in ihrer Funktion die EBFP anwenden / umsetzen / leben, können Sie mir beschreiben, wie sie eine EBF praktizieren / umsetzten / leben?
   Unterfragen
    a. Was bedeutet EBN für Sie?
    b. Welche Strategien wenden Sie an, um EBN in Ihre/deine Pflegepraxis umzusetzen (Evidenz-Praxis-Transfer)
    c. Wenn APN-Rolle:
        i. Können Sie mir von Ihrer/deiner Forschungstätigkeit berichten? Wie kann ich mir das vorstellen (bspw. Mitarbeit an klinikinternen Guidelines, SOPs etc.)?
        ii. Können Sie mir aus Ihrer Pflegeentwicklungstätigkeit berichten!
            1- Entwickeln Sie Pflegeinterventionen?
            2- Wie setzen Sie diese in die Pflegepraxis um?

Vielen Dank, dann sind wir am Ende des Interviews angekommen. Gibt es noch etwas, dass Sie gerne ergänzen möchten?

Eine abschliessende Frage: Dürfen wir Sie für ein Folgeinterview zu einem späteren Zeitpunkt anfragen (falls ja, private Mailadresse erfragen)?

3.3 Themenblock i2 (nach t3, 12 Monate nach Studienabschluss)

Einstiegsfrage: Erzählen Sie bitte, wie Ihre aktuelle berufliche Tätigkeit aussieht.

- Wenn in einer APN-Rolle: gleich wie bisher oder neu übernommen (seit dem letzten Interview)?
    a. ggf. berufliche Veränderungspläne umgesetzt?
- Welche Kompetenzen können Sie in Ihrer derzeitigen Tätigkeit ausüben?
- Welche Hauptaufgaben haben Sie?
- In welchem Fachgebiet und Setting sind Sie tätig? (Fragen werden teilweise bereits bei den «Demografics» erhoben)

Infos für Interviewführende Person: Der APNCAI beinhaltet 8 Dimensionen davon werden während dem Interview i1 und Interview i2 jeweils 4 Dimensionen erhoben (gelb Interview i1 / türkis Interview i2).

1. Evidenzbasierte Forschung und Praxis
2. Klinische und fachliche Führung
3. Berufliche Selbständigkeit
4. Interprofessionelle Beziehungen und Mentoring
5. Qualitätsmanagement
6. Pflegemanagement
7. Ausbildung und Berufsbildung
8. Gesundheitsförderung

Dimension 3: Berufliche Selbständigkeit (professional autonomy)

Hamric et al. (2013) beschreibt bei der APN-Rolle die berufliche Selbständigkeit. (Übergeordneter Fokus: In welchen Bereichen werden welche Ergebnisse erzielt und was berichten andere über diese Selbständigkeit?)

1. Können Sie mir beschreiben, in welchen Bereichen Sie selbständig entscheiden und agieren können (bezogen auf Aufgabenbereich / Themenbereiche)?
    a. Was bedeutet die Selbständigkeit für Sie?
    b. Wo könnte die Selbständigkeit noch ausgebaut werden, was könnten von Ihnen noch übernommen werden?
2. Tragen Sie Eigenverantwortung und wie sieht diese Verantwortung aus?
    a. Was macht das mit Ihnen?
    b. Und mit Ihren Kolleg:innen?
    c. Und mit den von Ihnen betreuten Patient:innen?

Übergang Dimension 4 (Interprofessionelle Beziehungen und Mentoring): Sie tragen Verantwortung, mit wem teilen Sie diese?

Dimension 4: Interprofessionelle Beziehungen und Mentoring

Die APNs arbeiten kollaborativ und übernehmen Brückenfunktionen. Diese zeichnet sich durch ein interprofessionelles miteinander aus.

1. Zu welchen Themen arbeiten Sie mit anderen Berufsgruppen zusammen?
    a. Und wie sieht diese Zusammenarbeit aus?
    b. Wie wird in dieser Zusammenarbeit kommuniziert?
2. Zu welchen Themen arbeiten Sie Intraprofessionell zusammen
    a. Und wie sieht diese Zusammenarbeit aus?
    b. Wie wird in dieser Zusammenarbeit kommuniziert
3. Was denken Sie, wie würden Ihre Kolleg:innen die Zusammenarbeit mit Ihnen beschreiben.

Dimension 5: Qualitätsmanagement

Die Qualitätsmanagementfunktion (QM) ist ebenfalls Teil der APN Rolle.

1. Was / welche Aufgaben übernehmen Sie im Rahmen des QM?
    a. bezogen auf das systemische (fachliche) (bspw. Implementation von Leitlinien) und
    b. Personenbezogene QM (institutsinterne WB/Schulungen/Sicherung von Versorgungsstrukturen)?
2. Wie kann ich mir das an einem Beispiel konkret vorstellen?

Dimension 6: Pflegemanagement (Leadership)

Überleitung: Wie wechseln zur Thematik Leadership. Falls es sich um die/den gleichen Teilnehmenden handelt kann ein Bezug vor einem Jahr hergestellt werden – damals haben wir uns schon über dieses Thema unterhalten.

1. Das Hamric Modell et al. (2013) sieht eine Leadership-Funktion vor, können Sie mir erzählen, wie Sie diese Funktion ausüben?
    a. Welche Erfahrungen haben Sie zu/mit Leadership gemacht?
    b. Beschreiben Sie mir, wie Sie Leadership in Ihrem Berufsalltag erleben und praktizieren/umsetzen!

Vielen Dank, dann sind wir am Ende des Interviews angekommen. Gibt es noch etwas, dass Sie gerne ergänzen möchten?

Eine abschliessende Frage: Dürfen wir Sie für ein Folgeinterview zu einem späteren Zeitpunkt anfragen (falls ja, private Mailadresse erfragen)?

3.4 Themenblock i3 (vor t4, mehr als 12 Monate und bis zu 3 Jahre nach Studienabschluss)

Themenblock A – Ausübung der APN-Rolle (und Rollenidentifikation – was und wie, Praktiken, Routinen, Zusammenarbeit mit anderen Professionen, Kommunikation)

Einstiegsfrage:

1. Beschreibe mir deinen APN-Alltag!

    a. Wie verläuft ein normaler Arbeitstag von dir?
    b. Wie ist dein heutiger Tag verlaufen?

2. Wie würdest du einer Studierenden im Praktikum (bspw. Workshadowing) beschreiben wie du deine APN-Funktion ausübst (Hauptaufgaben / Tätigkeitbereiche / Aufgabengebiete)?

3. Was denkst du, wie beschreiben bspw. Mediziner:innen dein Aufgabengebiet (oder andere Professionen wie Physio etc.)?

4. Wie sehen dich die von dir betreuten PatientInnen?

5. Kannst du mir bitte beschreiben, wie du dir zukünftig deinen Arbeitsbereich als APN vorstellst?

    a. Welche Arbeitsbereiche / Aufgaben könnten APNs auch noch übernehmen/ abgeben?
    b. Wie hat sich dein Bild von einer APN verändert in der letzten Zeit bzw. seit du diese Rolle ausübst?

6. Möchtest du zu diesem Thema noch etwas ergänzen, bevor wir zum nächsten Thema wechseln?

Themenblock B – Entwicklung der Kompetenzen/Fähigkeiten (Skills)

Überleitung: Sprechen wir noch über deine/Ihre persönliche Entwicklung.

1. Denke zurück an deinen Start/Beginn hier als APN, du bist xy Jahre hier. Erzähle mir von deinem Einstieg als APN!
    a. Hast du dafür dein berufliches Setting verändert? (bspw. Stellenwechsel)
    b. Was ist seit damals neu dazugekommen?
        i. Hast du spezifisch Fähigkeiten für diese APN-Rolle erlernt, die du vorher nicht konntest / beherrschst hast?
    c. Was machst du jetzt nicht mehr?
        i. Fehlen dir diese?

2. Wie sieht für dich eine ideale APN-Rolle in der Schweiz aus?
    a. Wie sieht der zukünftige Tätigkeitsbereich einer APN in der Schweiz aus. Gibt es noch etwas, dass du für die jetzigen Tätigkeiten einer APN erlernen würdest?
    b. Wieso war es bisher noch nicht möglich?

3. Möchtest du zu diesem Thema noch etwas ergänzen, bevor wir zum nächsten Thema wechseln?

Themenblock C – Vorbereitung auf APN-Rolle durch Studium (Kompetenzentwicklung / Ziel Studium evaluieren / weiterentwickeln)

Überleitung: Sprechen wir noch über dein zurückliegendes Studium.

1. Denke an deine Studienzeit zurück. Wie hat dich dein Studium auf deine APN-Rolle vorbereitet?
    a. Was ist dir als **förderlich** in Erinnerung geblieben?
        i. Wobei hast du das Studium als Unterstützung / hilfreich erlebt?
    b. was hat dir für deine heutige APN-Rolle / heutiges Aufgabengebiet **gefehlt**?

   c. Welche Fähigkeiten hast du dir **selbst angeeignet / nachgearbeitet**?
   d. Was hast du **nicht anwenden / umsetzen** können?

2. Wie würdest du ein APN-Studium gestalten? (Fokus: Vorbereitung auf Rolle)
   a. Welche Lernformen (eLearning/Präsenz)
   b. Welche Inhalte (Modulen)
   c. Praxistransfer(s) (Praktika, Workshadowing)
   d. Kultur (Lehrperson / Studierende, Work-Life-Balance, Vereinbarkeit Beruf/Studium)

3. Möchtest du zu diesem Thema noch etwas ergänzen, bevor wir zu den Abschlussfragen wechseln?

<u>Abschliessende Fragen (Selbsteinschätzung / Bryant-Lukosius et. al.; 2016)</u>

Abschlussfrage Verortung: Wo siehst du dich heute in Bezug auf die Ausübung deiner APN-Rolle?

1. Wohin möchtest du dich selbst hin entwickeln? (CNS – NP-Kontiuum) / Zukunftspläne?

Figure 1. Continuum of advanced practice nursing roles

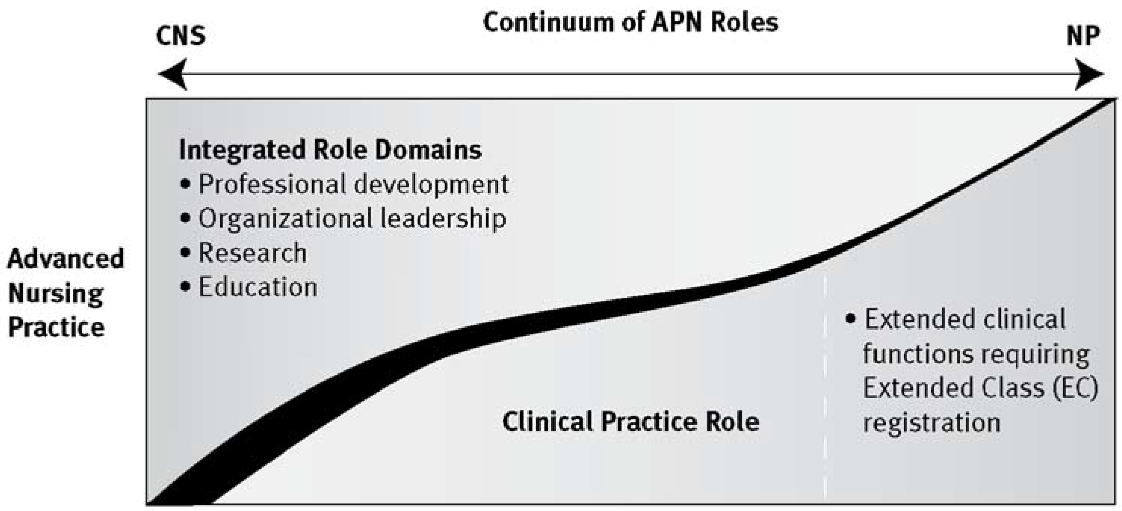

CNS= Clinical nurse specialist
Bryant-Lukosius, D. 2004 and 2008. *The Continuum of Advanced Practice Nursing Roles.* Unpublished document.
(Bildquelle: APN Kontinuum)

3.5 Abschluss: Offene Fragen oder Anmerkungen, Dank und Verabschiedung

Nun sind wir am Ende unseres Interviews angekommen. Gibt es von deiner/Ihrer Seite noch Punkte, die du/Sie als wichtig erachten und nicht platzieren konnten?

Eine abschliessende Frage: Dürfen wir Sie für ein Folgeinterview zu einem späteren Zeitpunkt anfragen (falls ja, private Mailadresse erfragen)?

Vielen Dank für deine/Ihre Zeit und Ihre Informationen.

Interviewnachbereitung

- Feldprotokoll, Demografics und Selbsteinschätzung CNS-NP-Kontinuum vervollständigen
- Datenbeschriftung (yyyymmtt-Hochschule-IDxy) bspw. 20220829-OST-ID01)
- Datensicherung: SwitchDrive (Folder Datensammlung / Daten / Analyse). Im Folder **Daten** neuen Folder mit allen Dokumenten erstellen (Beschriftung: ZHAW_ID01)

Literaturverzeichnis

Creswell, J. W., & Clark, V. L. P. (2017). *Designing and conducting mixed methods research*. Sage publications.

Hamric, A. B., Hanson, C. M., Tracy, M. F., & O'Grady, E. T. (2013). *Advanced Practice Nursing-E-Book: An integrative Approach* (5th Edition). Elsevier Health Sciences.

Helfferich, C. (2014). *Die Qualität qualitativer Daten* (Vol. 4). Wiesbaden: VS Verlag für Sozialwissenschaften.

Kelle, U. & Kluge, S. (2010). *Vom Einzelfall zum Typus.* Fallvergleich und Fallkontrastierung in der qualitativen Sozialforschung (Qualitative Sozialforschung, Bd. 15, 2., überarbeitete Auflage). Wiesbaden: VS Verlag für Sozialwissenschaften.

Kuckartz, U. (2014). *Mixed methods: methodologie, Forschungsdesigns und Analyseverfahren*. Springer-Verlag.
